# Insights into Occlusal Analysis: Articulating Paper versus Digital Devices

**DOI:** 10.3390/jcm13154506

**Published:** 2024-08-01

**Authors:** Manuela-Maria Manziuc, Mara Mihaela Savu, Oana Almăşan, Daniel-Corneliu Leucuţa, Manuela Tăut, Cosmin Ifrim, Denisa Berindean, Andreea Kui, Marius Negucioiu, Smaranda Buduru

**Affiliations:** 1 Prosthetic Dentistry and Dental Materials Department, Iuliu Hațieganu University of Medicine and Pharmacy, 32 Clinicilor Street, 400006 Cluj-Napoca, Romania; 2 Department of Medical Informatics and Biostatistics, Iuliu Hatieganu University of Medicine and Pharmacy, 400349 Cluj-Napoca, Romania

**Keywords:** digital dentistry, occlusal contacts, OccluSense, intraoral scanner, articulating paper

## Abstract

**Background:** As the demand for digital dentistry constantly increases, digital devices are gradually replacing conventional methods of recording occlusal contacts. The study aimed to assess the inter-rater reliability of occlusal contact point detection using 40 μm articulating paper, Medit i700, and OccluSense and to compare the distribution of occlusal contacts using the articulating paper and intraoral scanner. **Material and Methods:** The study included 25 participants aged 20 to 30 (13 women and 12 men). Photographs of contact points were taken and marked in maximum intercuspal position (MIP), in protrusive and laterotrusive movements, on working and non-working sides using 40 μm articulating paper and digital devices. The Cohen’s Kappa coefficient assessed the inter-rater reliability. The Wilcoxon signed-rank test was used to compare dependent groups, articulating paper, and Medit i700. **Results:** The Cohen’s Kappa index showed that almost perfect agreement was achieved with 40 μm articulating paper. Compared to Medit i700, the 40 μm articulating paper showed an increased mean number of contacts per tooth, except for the third molars. **Conclusions**: The 40 μm articulating paper has detected more overall contacts than the digital devices, particularly in the posterior areas. An ideal method for registering occlusal contacts has not been established yet.

## 1. Introduction

When creating complex oral rehabilitations, achieving functional integrated dental restorations requires precisely recording static and dynamic occlusal relationships [1]. As the demand for digital dentistry constantly increases, new digital devices are gradually replacing conventional methods of recording occlusal contacts [2,3]. However, the debate on the most accurate method of recording the occlusal relationship is still open since there are no authentic “gold standard” criteria to assess the accuracy and reliability of different occlusal diagnostic tools [4,5].

Traditionally, clinicians use articulating paper to mark and subjectively evaluate the distribution and intensity of tooth contacts during mandibular functional movements or parafunction [6]. The sensitivity and reliability of articulating paper are related to the strength and elasticity of the material, such as paper, polyester, or silk, and its thickness, which may vary from 8 to 200 microns (μm) [7]. Conventionally, thicker articulating paper marks are broader and may have different numbers of occlusal contacts, while the thinner one highlights more precisely their number and distribution [8]. The clinician should also acknowledge the limitations of this widely used diagnostic tool, such as the impossibility of evaluating the bite sequence and dental contact pressure level, the saliva moisture, which may determine false negative or positive occlusal contacts, and the patients need to perform several opening and closing movements to assess the occlusal contacts [9].

Lately, the development of digital technology has revolutionized the dentistry field, allowing us to evaluate with higher precision the occlusal contacts, both in static and dynamic occlusion [10]. OccluSense (Dr. Jean Bausch, GmbH & Co KG, Köln, Germany) is a new occlusal analysis system for recording dental relationships. It consists of a sensor covered in articulating paper which is inserted into the handheld, which records and transmits the data to the OccluSense-iPad-App (Apple Inc., Cupertino, CA, USA). Unlike conventional methods, OccluSense also records the precise moment of the dental contact besides the distribution and intensity of the occlusal force [11]. OccluSense utility was only tested for marking contacts with ink, which means any accuracy or agreement about its functionality as a digital occlusion tool was not reported. OccluSense was simply being tested for its’ 60 μm ink marking ability [11].

However, a main aspect that should be considered when assessing the accuracy of the digital occlusal contacts’ force is represented by the characteristics of the force-jump gradient per color. OccluSense uses 4-color gradients and heights to represent 256 force levels, each color change corresponds to 64 force levels, leading to significant difficulty in detecting subtle differences in occlusal contact forces between adjacent contacts [11]. On the other hand, the T-Scan system displays 18-color gradients to assess 256 force levels, resulting in 14 force levels per color with the capacity to detect more precise and subtle differences between the occlusal forces of the adjacent teeth [11].

In their study, Popa et al. [12] demonstrated that compared to the Medit i600 intraoral scanner, the T-Scan III provides more accurate data regarding the distribution, surface area, amplitude, and dynamic over time of the occlusal contacts. In MIP, both digital systems recorded the highest numbers of dental contacts, with significant differences between the surface of the contacts. According to Bostancıoğlu et al. [13], T-Scan showed higher sensitivity in the diagnosis of both light and tight occlusal contacts, compared to CEREC Omnicam, which had a good sensitivity in assessing the tight dental contacts. In another study [14], the researchers demonstrated the T-Scan system’s ability to evaluate the strength of the occlusal contacts, regardless of their area, while the 3Shape system marks contacts based on proximity between each tooth. Kerstein et al. [15] concluded that the T-Scan system was very precise in assessing the occlusal contacts on both the left and right sides and the acquired force percentage was imbalanced, but highly repeatable, at various intercuspation force levels.

OccluSense is a digital occlusion instrument that captures occlusal contact force and time. OccluSense cannot discriminate between contact pressures on incisal edges, occlusal surfaces, cingulums, mesial and distal marginal ridges, fossa, and cusp tips. OccluSense has no defined dental arch. It is a general arch shape with no delineations for particular tooth shapes, tooth forms, marginal ridges, cusps, and so on. If a recorded patient is missing some teeth, no teeth may be removed from the OccluSense arch [11].

The intraoral scanners (IOSs) record the contacts in static and dynamic occlusion and can also determine the occlusal contact area [2,16]. Although the intraoral scanners (IOSs) and OccluSense (Dr. Jean Bausch, GmbH & Co KG) are digital devices used to enhance the clinical workflow, their reliability for registration and analyzing the static and dynamic occlusal relationship is still uncertain [11,17]. These devices may provide more precise and objective data, eliminating clinician subjectivity and enhancing the refinement of static and dynamic occlusal adjustments [8]. Yet, there is insufficient data to compare the accuracy of occlusal relationship records with conventional and newly introduced digital devices [18,19].

This clinical study aimed at determining the inter-rater reliability of the static and dynamic occlusal recordings performed using 40 μm articulating paper (Dr Jean Bausch GmbH & Co KG, Koln, Germany), the intraoral scanner, Medit i700 (Medit i700, MEDIT, Seoul, Republic of Korea) and a digital occlusal analysis system, OccluSense system (Bausch, Dr. Jean Bausch GmbH & Co KG, Köln, Germany) and to compare the distribution of the static occlusal contacts registered using the intraoral scanner and articulating paper.

The null hypotheses were: (1) the static and dynamic occlusal recordings performed using articulating paper, Medit i700, and OccluSense would show similar inter-rater reliability; (2) the number of static occlusal contacts assessed per tooth, registered using an intraoral scanner and the articulating paper would be similar. The second null hypothesis was assessed only using articulating paper and Medit i700 since the color distribution of the occlusal contacts recorded by OccluSense is shown by a unique dental arch view, which displays the distribution and intensity of the occlusal status recorded between the upper and lower arch.

## 2. Materials and Methods

The study included 25 participants (13 women and 12 men), ranging in age from 20 to 30 years. The sample size met the criteria set by the estimation program (G*Power version 3.1.9.2, Franz Faul, Universität Kiel, Germany) [20]. For a Wilcoxon signed-rank test to compare the number of contacts per tooth to detect an effect size of 0.8, with a significance level of 5% and a power of 95%, required a minimum sample size of 24 participants. All the participants were students of the Faculty of Dental Medicine, “Iuliu Hațieganu” University of Medicine and Pharmacy, Cluj-Napoca, Romania. After receiving comprehensive information on the purpose and methodology of the study, each participant completed and signed the informed consent approved by the University’s Ethical Committee (approval number 53/21.03.2024).

This research included participants with complete dental arches up to the second molar with stable and functional occlusal relationships and healthy temporomandibular joints without clicking or pain. The exclusion criteria were considered anterior or posterior open bite, partial edentulism, dental wear, and signs or symptoms of temporomandibular disorders.

For each participant, one experienced operator (M.M.M) photographed the contact points marked in maximum intercuspal position (MIP) during protrusion and right and left laterotrusion on both the working and non-working sides using the 40 μm articulating paper. The intraoral scanner (Medit i700, MEDIT, Seoul, Republic of Korea) and the occlusal analysis system (OccluSense, Dr. Jean Bausch, GmbH & Co KG) made the digital scan recordings. Occlusal analysis and registration sessions were conducted one week apart for each participant to minimize the risk of muscle fatigue and ensure unbiased results. two operators (M.M.M., M.M.S.) independently analyzed the acquired data.

### 2.1. Articulating Paper

**Figure 1:**
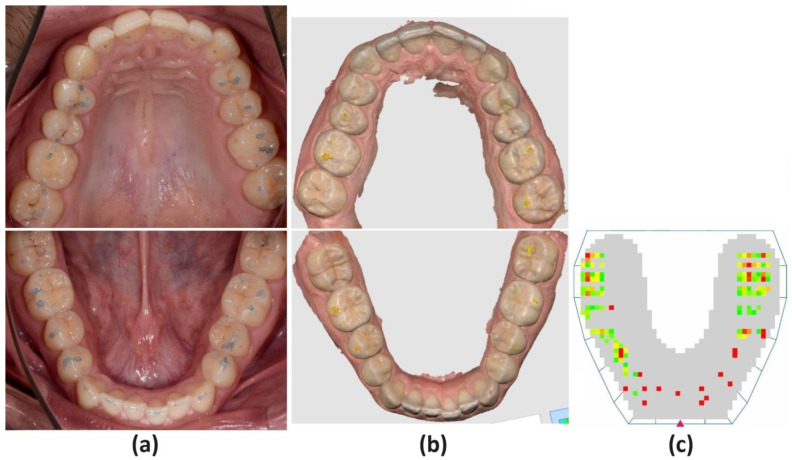
Images of maximum intercuspation position with the (**a**) 40 microns articulating paper, (**b**) Medit i700, (**c**) OccluSense independently evaluated by the researchers as dichotomous variables, coding the presence of occlusal contact, not its intensity.

The occlusal contacts were marked with 40 μm articulating paper (Dr Jean Bausch GmbH & Co KG, Koln, Germany). The participant was seated comfortably in a chair whose headrest properly supported his head. The patient was asked to perform opening and closing movements, protrusion, and laterotrusion movements to the right and left as the operator examined each type of movement and certified that the participant performed them correctly. The occlusal surfaces were well-dried using the air blower. Two tapes of blue articulating paper, retained by the articulating tape forceps, were simultaneously applied between the left and right sides of the dental arches, and the participant performed the opening and closing movements to mark the contact points in MIP (Figure 1a).

**Figure 2:**
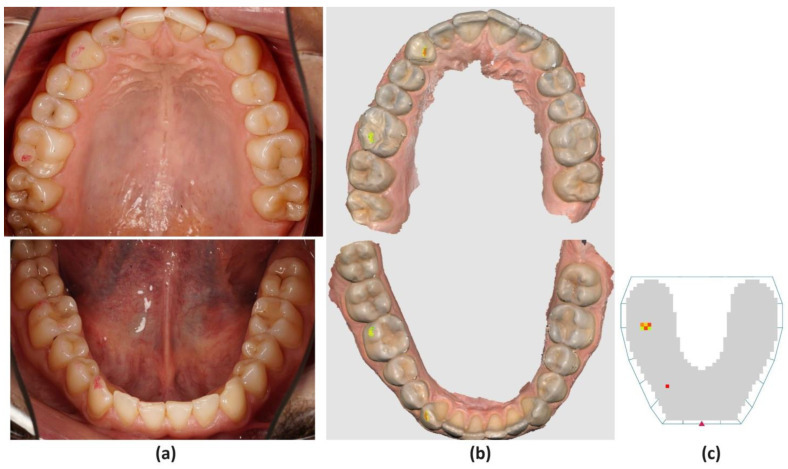
Images of active laterotrusive interference (ALI) during right lateral movement with the (**a**) 40 microns articulating paper, (**b**) Medit i700, (**c**) OccluSense. Evaluation of the presence and distribution of the contact points on the occlusal surfaces of the teeth.

Red horseshoe-shaped articulating paper was used to assess the protrusion movement. The guidance was considered functional when the central incisors, alone or with the lateral incisors or canines, guided the movement equally through the marginal ridges. The canines, alone or with the bicuspids and molars (group function), performed the laterotrusion movement. The working and non-working interferences or premature contacts for both functional guidances were assessed (Figure 2a). In the study, interference in any nonfunctional occlusal contact between antagonistic teeth during protrusion or laterotrusion and defined premature contact was considered as a nonfunctional contact between the antagonistic teeth at the end of the functional movements. Active interferences or premature contacts on the working side and passive ones on the non-working side were analyzed. After each examination of the static and dynamic occlusal contacts, the same operator used the cheek retractor, occlusal photo mirror (Doctoreyes GmbH, Ochsenhausen, Germany), and a digital camera (D7200 camera with 105 mm lens, Nikon Corporation, Tokyo, Japan) to take occlusal photographs of both the maxillary and mandibular arches.

### 2.2. Intraoral Scanner (IOS)

Before the scanning procedure, a lip retractor (OptraGate, Ivoclar Vivadent, Schaan, Liechtenstein) to enhance the visibility of the dental arches was used, and the dental surfaces were well air-dried. Optical scans of the maxillary and mandibular dental arches with an intraoral scanner (Medit i700, MEDIT, Seoul, Republic of Korea) were performed according to manufacturer instructions. The occlusal relationship was assessed by asking the participant to close the mouth in MIP and maintain dental contact. The intraoral scanner was positioned in the area corresponding to the canine and first molar, on the right and left sides, being orientated perpendicular to the occlusal plane, moved from posteriorly towards anteriorly, in a slightly ascending to descending direction. The maxillary and mandibular digital models were identified and brought in proper alignment in maximum intercuspal position by the Medit software program (Medit Scan for Clinics v3.3.0, MEDIT, Seoul, Republic of Korea). Afterward, the patient performed the previously mentioned mandibular movements recorded by the software program to assess the dynamic occlusal relationship. The Medit Occlusion Analyzer (Medit Scan for Clinics v3.3.0, MEDIT, Seoul, Republic of Korea) function analyzed the distribution of the dental contact points in MIP, and the Mandibular Movement Mode (Medit Scan for Clinics v3.3.0, MEDIT, Seoul, Republic of Korea) function assessed the occlusal contacts during protrusion and laterotrusion. The intensity of the dental contacts was set at 40 μm, corresponding to the thickness of the articulating paper, and represented as a colored map (Figure 1b and Figure 2b).

### 2.3. OccluSense

The occlusal analysis with the OccluSense (Bausch, Dr. Jean Bausch GmbH & Co KG, Koln, Germany) system was performed using the same protocol to prepare the dental arches for occlusal registration, as previously mentioned. The horseshoe-shaped OccluSense Sensor, 60 μm thick, consists of a printed electronic circuitry coated in red-colored ink. Occlusal registrations according to manufacturer instructions were performed and one sensor for each patient was used to record static and dynamic occlusal contacts. The operator carefully positioned the printed red triangle of the sensors corresponding to the lower interincisal line and successively recorded dental contacts in MIP and occlusal relationship in protrusion and laterotrusion, and the acquired data were processed by the OccluSense iPad App (Apple Inc., Cupertino, CA, USA) and displayed in a combined view. The two-dimensional view (2D) showed the dental contacts as colored squares. The sensor is a 60 μm thin foil that contains a printed circuit with 1018 pressure-sensitive pixels. On the other hand, the three-dimensional view (3D) represented the intensity of the occlusal pressure of each dental contact as bar diagrams, with different color gradients and heights corresponding to the intensity of the occlusal pressure. However, the OccluSense system uses 4-color gradients and heights to represent 256 force levels. Each color change corresponds to 64 force levels, leading to significant jumps between colors. Thus, the OccluSense system presents issues in accurately detecting subtle differences in occlusal contact forces between adjacent contacts [11] (Figure 1c and Figure 2c).

The OccluSense system provides a unique view of both dental arches, displaying the distribution and intensity of the occlusal contacts recorded between the upper and lower arch.

The data collection was performed and analyzed independently by two experienced, calibrated, and trained operators (M.M.M and M.M.S.) who assessed the intraoral occlusal photographs, the occlusal view of the intraoral scanners, and the occlusal analysis device in MIP, protrusion, and left–right laterotrusion on both working and non-working sides. Thus, the active propulsive interferences (API), active laterotrusive interferences (ALI), passive propulsive interferences (PPI), passive laterotrusive interferences (PLI), active propulsive premature contact (APPC), active laterotrusive premature contacts (ALPC), passive propulsive premature contacts (PPPC) and passive laterotrusive premature contact (PLPC) were analyzed. To determine the inter-rater reliability of the digital devices and articulating paper, the contact points were classified in a dichotomy variable, meaning the presence or absence of contact per tooth, regardless of the surface amount or contact intensity. The presence of any contact, no matter the intensity or its surface, was considered as the presence of contact, irrespective of, in some cases, whether from an artifact or not. OccluSense utility was only tested for marking contacts. The functionality of it as a digital occlusion tool was not assessed in this study. OccluSense was simply being tested for its’ 60 micron ink marking ability. To precisely measure the number of static occlusal contacts per tooth, registered using Medit i700 and the articulating paper, the most representative anatomical elements of each tooth structure were considered: the incisal edge and the occlusal surface, cingulum, mesial and distal marginal ridges or fossa, and cusp. All the teeth present on the arch were examined, including the second and third molars.

Statistical analysis was performed using Cohen’s Kappa coefficient to assess the inter-rater reliability of two raters that assessed each method separately. The Wilcoxon signed-rank test to compare dependent groups represented by articulating paper and the intraoral scanner was used. A statistical software program (R version 4.1.2; R Foundation for Statistical Computing) computed all statistical analyses.

## 3. Results

For each method, 40-μm articulating paper (Bausch), OccluSense (Bausch) and intraoral scanner (Medit i700), were used to assess the distribution of the contact points individually by each examinator. The Cohen’s Kappa index showed values of almost perfect inter-rater agreement for the 40 μm articulating paper, OccluSense, and intraoral scanner, with almost all values being above 0.9 (*p* < 0.05). The reliability for the 40 μm articulating paper showed almost perfect agreement, represented by the value 1, followed in decreasing order by the intraoral scanner and OccluSense for all the static and dynamic contacts (Table 1).

Except for the third molars, the mean number of contacts was higher per tooth for the 40 μm articulating paper than for the Medit i700, and the median number of contacts was either higher or equal for the articulating paper in contrast to the Medit i700. Significant differences regarding the number of contacts per tooth were observed between the 40 μm articulating paper and the Medit i700, except for the upper central incisors (1.1, 2.1), second (1.7, 2.7, 3.7, 4.7) and third molars (1.8, 2.8, 3.8, 4.8), but also lower lateral incisors (3.2), (Table 2).

The teeth were grouped by maxillary and mandibular anterior or posterior areas. The mean number of contacts was higher per tooth for the 40 μm articulating paper than for Medit i700 (Table 3) in each area. On average, between 0.5 and 1 more contact was observed by the 40 μm articulating paper compared to Medit i700.

## 4. Discussion

Noninvasive and predictable digital devices are widely used to record and analyze static and dynamic occlusal relationships [21,22]. Even though there is no consensus on the reference method for analyzing occlusal status, the articulation paper still remains widely used when assessing and comparing new digital devices [4,5].

Fraile et al. [23] investigated the reliability of the static occlusal contacts recorded using 8 μm articulating paper, the IOS (Trios Color POD, Phibo, 3 Shape, Copenhagen, Denmark), and T-Scan III (Tekscan, Boston, MA, USA). For the 8 μm articulating paper, their results indicated better inter-rater agreement (70.6%). The influence of the articulating paper thickness and the digital sensors used to evaluate the occlusal relationships can explain these findings. Regardless of the thickness of the sensors, 60 μm for OccluSense and 100 μm for T-Scan III, the articulating paper showed higher inter-rater agreement. Digital devices provide more precise and accurate information regarding the timing and intensity of occlusal contacts, but the data require more objective interpretation from clinicians, which leads to inconsistent results. Thinner articulating papers, 8 or 40 μm thick, or thinner sensors are more flexible than thicker ones and are better adapted to occlusal morphology, providing a more accurate distribution of occlusal contacts. Thus, the first hypothesis was accepted, since the inter-rater reliability for the 40 μm articulating paper and the two studied digital devices showed almost perfect agreement, ranging between 0.836 and 1. The highest inter-rater reliability was assessed for the 40 μm articulating paper, followed by Medit i700 and OccluSense for all the static and dynamic contacts. Therefore, our study confirms that both OccluSense and the Medit i700 intraoral scanner are highly reliable for detecting occlusal contacts, as Cohen’s Kappa values indicated almost perfect agreement (above 0.9, *p* < 0.05). This result may be explained by the clinician’s higher experience, ease of interpretation, and consistency in the use of the articulating paper, compared to digital devices, which require a learning curve to achieve uniformity in assessing the virtual occlusal relationships.

The second null hypothesis was rejected since the number of static occlusal contacts assessed per tooth registered with both digital devices and the articulating paper was not similar.

Even though intraoral scanners are used extensively to take impressions of the dental arches, their capacity to record the occlusal contacts is still uncertain [24]. However, the new IOS software, such as the Mandibular Movement Mode (Medit Scan for Clinics v3.3.0, Medit, Seoul, Republic of Korea) function, can record static and dynamic occlusal status. 

Medit i700 shows excellent performance in capturing three-dimensional (3D) images of the dental arches and occlusal surfaces and also allows the assessment of occlusal relationships. Revilla-Leon et al. [17] concluded that an IOS system may assess the static occlusal status like a conventional technique when the dental arches are complete, the occlusal contacts are stable, and there is a single prepared or missing tooth. Other important factors that influence the accuracy of the IOSs may also be the number, location, and dimension of the virtual contacts and the length of the dental arches. For higher accuracy of the virtual recording, it is necessary to scan a bilateral static occlusal relationship involving four teeth when reaching the MIP position [25].

Yet, there is limited data regarding the accuracy of virtual occlusal contacts assessed by IOS software [22,26,27].

A higher number of static occlusal contacts were registered with the 40 μm articulating paper compared to Medit i700, except for the upper central incisors (1.1, 2.1), second (1.7, 2.7, 3.7, 4.7) and third molars (1.8, 2.8, 3.8, 4.8), but also lower lateral incisors (3.2), both on the right and left side. Also, significant differences were found between the upper and lower tooth groups and mandibular and maxillary anterior and posterior groups. On average, the 40 μm articulating paper recorded between 0.5 and 1 more contact than Medit i700.

The position of the teeth on the dental arch, the anatomy of the occlusal surface, the scanning procedure, and the characteristics of the IOS software may explain the exceptions. Several studies yielded similar results, stating that the IOS recorded a matching number of occlusal contacts compared to articulating paper [17,25,28,29] due to the higher accuracy of 3D occlusal images than of intraoral assessment of occlusal contacts. However, no consensus was reached regarding the reference method when analyzing the occlusal relationship. Therefore, the articulating paper is still considered a reference method, even though there is a lack of information regarding its accuracy and precision, and the saliva moisture, the bite sequence, and dental contact pressure level may determine false negative or positive occlusal contacts, which are subjectively evaluated by the clinician [9]. To standardize the thickness of the occlusal interposed medium, the 40 μm-thick articulating paper was employed to compare it to IOS occlusal registration. Nevertheless, assessing the occlusal contacts with high accuracy is a demanding but mandatory clinical procedure, which may lead to precise diagnosis and treatment planning, improving treatment outcomes.

The limitations of this study are the difference in thickness between the OccluSense sensor (60 μm) and the articulating paper (40 μm) and Medit i700 (40 μm) and the lack of standardization of the occlusal registration methods. Further, it is well known that when doctors subjectively interpret ink markings, errors in interpretation can occur. This subjectivity may affect the reliability of this study’s reported results [30,31,32]. Nevertheless, to limit this bias in our study, any visible contact was marked as the presence of a contact, thus the intensity of the contact did not influence the choice. Despite the impressive development of digital dentistry, there is no ideal method for occlusal assessment.

The originality in this study consists of assessing inter-rater reliability concerning the dynamic functional or non-functional occlusal contacts during protrusive and laterotrusive movements evaluated both on the working and non-working sides.

Despite the impressive development of digital dentistry, there is no ideal method for occlusal assessment. There are advantages and disadvantages to both conventional and digital systems; accordingly, the clinician should use them complementarily rather than exclusively for an accurate and detailed examination. Nevertheless, patient proprioception is a key element to be considered in achieving a more comprehensive occlusal analysis.

Further research should be conducted to compare the number and distribution of static and dynamic occlusal contacts using different brands of intraoral scanners and digital occlusal analysis systems to integrate them properly into the digital workflow.

## 5. Conclusions

Both digital systems detected occlusal contacts to some extent, but the 40 μm articulating paper detected more overall contacts than either digital device, particularly in the posterior areas. However, an ideal method for registering occlusal contacts has not been established yet.

## Figures and Tables

**Table 1:** Cohen’s Kappa inter-rater agreement for the two examiners observing one registration method.

	40-μm Articulating Paper (Bausch)		OccluSense (Bausch)		Intraoral Scanner (Medit i700)	
	Cohen’s Kappa	*p*-Value	Cohen’s Kappa	*p*-Value	Cohen’s Kappa	*p*-Value
MIP	1	<0.001	1	<0.001	1	<0.001
P	1	<0.001	0.981	<0.001	1	<0.001
L	1	<0.001	0.992	<0.001	1	<0.001
API	1	<0.001	0.936	<0.001	0.979	<0.001
ALI	1	<0.001	0.966	<0.001	0.989	<0.001
PPI	1	<0.001	0.962	<0.001	1	<0.001
PLI	1	<0.001	1	<0.001	1	<0.001
APPC	1	<0.001	0.836	<0.001	0.946	<0.001
ALPC	1	<0.001	0.947	<0.001	0.976	<0.001
PPPC	1	<0.001	1	<0.001	1	<0.001
PLPC	1	<0.001	0.952	<0.001	1	<0.001

MIP (maximum intercuspal position); P (protrusion); L (laterotrusion); API (active propulsive interference); ALI (active laterotrusive interference); PPI (passive propulsive interference); PLI (passive laterotrusive interference); APPC (active propulsive premature contact); ALPC (active laterotrusive premature contact); PPPC (passive propulsive premature contact); PLPC (passive laterotrusive premature contact).

**Table 2:** Comparison of the number of contacts per tooth between 40 μm articulating paper and Medit i700.

Tooth	40 μm Articulating Paper, M (SD)	40 μm Articulating Paper, Med (IQR) [Range]	Medit.i700, M (SD)	Medit.i700, Med (IQR) [Range]	*p*
1.1	1 (0.78)	1 (0–2) [0–2]	0.75 (0.74)	1 (0–1) [0–2]	0.095
1.2	0.83 (0.76)	1 (0–1) [0–3]	0.38 (0.49)	0 (0–1) [0–1]	0.003
1.3	1.62 (0.88)	2 (1–2) [0–3]	0.79 (0.78)	1 (0–1) [0–2]	<0.001
1.4	2.26 (0.81)	2 (2–3) [0–3]	1.04 (0.71)	1 (1–1.5) [0–2]	<0.001
1.5	2.21 (0.83)	2 (2–3) [0–3]	1.21 (0.66)	1 (1–2) [0–2]	<0.001
1.6	2.92 (1.1)	3 (2–4) [1–5]	1.58 (1.02)	1.5 (1–2) [0–4]	<0.001
1.7	2.42 (1.1)	2.5 (2–3) [0–4]	1.96 (1.04)	2 (1–3) [0–3]	0.09
1.8	0.29 (0.69)	0 (0–0) [0–3]	0.46 (0.93)	0 (0–0.25) [0–3]	0.423
2.1	0.96 (0.75)	1 (0–1.25) [0–2]	0.71 (0.81)	0.5 (0–1) [0–2]	0.095
2.2	1.12 (0.68)	1 (1–2) [0–2]	0.79 (0.72)	1 (0–1) [0–2]	0.04
2.3	1.21 (0.72)	1 (1–2) [0–3]	0.5 (0.72)	0 (0–1) [0–2]	<0.001
2.4	2.25 (0.85)	2.5 (1.75–3) [1–3]	1.08 (0.97)	1 (0–2) [0–3]	<0.001
2.5	2.29 (0.69)	2 (2–3) [1–3]	0.71 (0.62)	1 (0–1) [0–2]	<0.001
2.6	3.29 (1.04)	3.5 (2.75–4) [1–5]	1.42 (1.02)	1 (1–2) [0–4]	<0.001
2.7	2.08 (0.88)	2 (1.75–3) [0–3]	1.96 (1.12)	2 (1–3) [0–4]	0.681
2.8	0.08 (0.28)	0 (0–0) [0–1]	0.25 (0.53)	0 (0–0) [0–2]	0.174
3.1	0.92 (0.65)	1 (0.75–1) [0–2]	0.58 (0.72)	0 (0–1) [0–2]	0.025
3.2	0.87 (0.46)	1 (1–1) [0–2]	0.65 (0.57)	1 (0–1) [0–2]	0.11
3.3	1.25 (0.61)	1 (1–2) [0–2]	0.71 (0.69)	1 (0–1) [0–2]	0.007
3.4	1.29 (0.46)	1 (1–2) [1–2]	0.83 (0.7)	1 (0–1) [0–2]	0.005
3.5	1.92 (0.78)	2 (1.75–2) [0–3]	1.04 (0.81)	1 (0–2) [0–2]	<0.001
3.6	3 (1.14)	3 (2–4) [0–5]	1.42 (1.06)	1 (1–2) [0–4]	<0.001
3.7	2.38 (1.24)	2 (2–3) [0–5]	2.12 (1.12)	2 (1–3) [0–4]	0.217
3.8	0.04 (0.2)	0 (0–0) [0–1]	0.17 (0.48)	0 (0–0) [0–2]	0.149
4.1	0.79 (0.59)	1 (0–1) [0–2]	0.58 (0.65)	0.5 (0–1) [0–2]	0.037
4.2	0.71 (0.55)	1 (0–1) [0–2]	0.38 (0.49)	0 (0–1) [0–1]	0.006
4.3	1.08 (0.41)	1 (1–1) [0–2]	0.71 (0.69)	1 (0–1) [0–2]	0.014
4.4	1.43 (0.66)	1 (1–2) [0–3]	0.87 (0.63)	1 (0.5–1) [0–2]	0.007
4.5	1.92 (0.58)	2 (2–2) [1–3]	0.96 (0.69)	1 (0.75–1) [0–2]	<0.001
4.6	2.92 (1.02)	3 (2–4) [1–5]	1.79 (1.02)	2 (1–2) [0–4]	<0.001
4.7	2.46 (1.1)	2.5 (1.75–3) [1–4]	1.79 (1.06)	2 (1–3) [0–3]	0.068
4.8	0.38 (1.01)	0 (0–0) [0–4]	0.42 (0.83)	0 (0–0.25) [0–3]	0.86

M, mean; SD, standard deviation; Med, median; IQR, interquartile range; range, [minimum–maximum].

**Table 3:** Comparison of the number of contacts per tooth grouped by maxillary and mandibular anterior or posterior areas between 40 μm articulating paper, and Medit i700.

	40 μm Articulating Paper, M (SD)	40 μm Articulating Paper, Med (IQR) [Range]	Medit.i700, M (SD)	Medit.i700, Med (IQR) [Range]	*p*
Mandibular anterior teeth	0.94 (0.57)	1 (1–1) [0–2]	0.6 (0.64)	1 (0–1) [0–2]	<0.001
Mandibular posterior teeth	1.77 (1.29)	2 (1–3) [0–5]	1.14 (1.04)	1 (0–2) [0–4]	<0.001
Maxillary anterior teeth	1.12 (0.79)	1 (1–2) [0–3]	0.65 (0.72)	1 (0–1) [0–2]	<0.001
Maxillary posterior teeth	2.01 (1.29)	2 (1–3) [0–5]	1.17 (1.03)	1 (0–2) [0–4]	<0.001

M, mean; SD, standard deviation; Med, median; IQR, interquartile range; range, [minimum–maximum].

## Data Availability

Data are available from the first author upon request.
